# Changes in oral health-related quality of life among Austrian preschool children following dental treatment under general anaesthesia

**DOI:** 10.1007/s00784-020-03598-6

**Published:** 2020-09-24

**Authors:** Sarra Boukhobza, Tanja Stamm, Johannes Glatthor, Nicola Meißner, Katrin Bekes

**Affiliations:** 1 Department of Paediatric Dentistry, University Clinic of Dentistry, Medical University of Vienna, Sensengasse 2a, 1090 Vienna, Austria; 2 Center for Medical Statistics, Informatics, and Intelligent Systems, Section for Outcomes Research, Medical University of Vienna, Spitalgasse 23, 1090 Vienna, Austria; 3 Salzburg, Austria

**Keywords:** Oral health-related quality of life (OHRQoL), Early Childhood Oral Health Impact Scale (ECOHIS), General anaesthesia, Oral rehabilitation

## Abstract

**Objectives:**

To analyse possible changes in oral health-related quality of life (OHRQoL) before and after dental treatment under dental general anaesthesia (DGA) among Austrian preschool children.

**Methods:**

A consecutive sample of 89 parents of children aged 2 to 5 years, suffering from early childhood caries (ECC) and scheduled for DGA, were recruited from two locations in Austria (Vienna and Salzburg). Parents self-completed the German version of the ECOHIS before (baseline) and 4 weeks (T4) after their child’s dental treatment. The ECOHIS consists of 13 questions and is divided into two main parts, namely, the child impact section (9 items) and the family impact section (4 items).

**Results:**

A total of 80 children (89%) completed a sufficient number ECOHIS questions at baseline and the follow-up assessment after 4 weeks. “Pain in the teeth, mouth, and jaws” and “difficulty eating some foods” from the child section and parents’ ratings of “feeling upset” and “guilty” were the most frequently reported impacts at baseline. The ECOHIS total score decreased significantly from a mean of 14.60 to 9.89 (*p* < 0.001) after DGA treatment, revealing a large effect size for the child (0.8) section, family (0.6) section, and the total score (0.8). Parents rated their child’s overall and oral health significantly higher after the DGA treatment (*p* < 0.001).

**Conclusions:**

Significant improvements in oral health-related quality of life were observed 4 weeks after DGA in children suffering from ECC.

**Clinical relevance:**

ECC has an impact on OHRQoL. Rehabilitation under general anaesthesia makes a sustainable improvement.

## Introduction

Despite improvements in efforts regarding dental health education, the percentage of children with decayed deciduous teeth remains startling high [1]. Early childhood caries (ECC) is still one of the most common diseases worldwide affecting 621 million children [2]. A recent representative study in Austria found that 45% of 5- to 6-year-old children have caries experience [3]. In addition to this quandary, the treatment of young children is often a challenging task. Although behaviour management techniques can be used to facilitate dental treatment, often dentists have to deal with little cooperative patients. Many children suffer of dental treatment-related anxiety or are difficult to treat/not compliant due to various other reasons [4]. Hence, the most common and efficient way of treating these patients is under general anaesthesia (GA) [5].

Nowadays, the concept of oral health-related quality of life (OHRQoL) has become important to assess oral health status in children and in adults [6] as clinical indicators alone do not reveal the full impact of oral conditions on the psychosocial wellbeing of a patient [7]. A common approach at this age is to ask the parents and potential other adult caregivers to complete the questionnaire. One of the most frequently used instruments to assess OHRQoL in preschool children is the Early Childhood Oral Health Impact Scale (ECOHIS), a questionnaire designed for adult caregivers, which was originally developed in the USA [8].

Without treatment, ECC is a fast progressing condition that can drastically affect a child’s quality of life, by leading to severe pain and other restrictions in daily life [9]. Even with the rise of attention regarding the importance and general effect of OHRQoL measurements, there are only few studies that use the ECOHIS as an instrument to evaluate the OHRQoL of children that received dental treatment under DGA [10], and none has so far been conducted in Austria. Given the prevalence of such treatment in young children, this presents a significant and problematic gap.

This study aims to close this gap of knowledge by evaluating changes in OHRQoL among Austrian preschool children aged 2–5 years before and after dental treatment under DGA using the German version of the ECOHIS [11]. The aim is to provide practitioners with more empirical evidence to evaluate the quality of life-related benefits of dental treatment of young children after dental treatment under DGA.

## Material and methods

A prospective, longitudinal, pre-posttest study was conducted in a consecutive sample of Austrian preschool children aged 2 to 5 years recruited at the Department for Paediatric Dentistry of the University Clinic of Dentistry in Vienna and a private practice in Salzburg.

### Subjects and setting

A consecutive sample of 89 primary caregivers of children aged 2 to 5 years (mean age 3.6, SD ± 1.1), suffering from ECC and awaiting oral treatment under DGA, were recruited over a period of 24 months. Eligible to the study were children who had early childhood caries that required treatment under DGA due to behavioural management issues. The enrolment into this study was voluntary. Extended information leaflets on the aim of the study were handed out and explained to the parents who gave their written and oral informed consent. A necessary requirement for inclusion into the study was fluency in German of the participants’ caregivers. The approval for the study procedures was granted by the ethics committee of the local University Review Board (Medical University of Vienna, #1822-2015).

All of the participants were provided with comprehensive counselling and clinical examination, consisting of thorough oral hygiene instructions and dietary advice before and after the treatment to ensure a long lasting positive effect and impact. Data of the examination, detailed diagnoses and treatment plan, as well as the duration of the DGA were protocolled. As a measurement of dental caries experience, we used the dmft-index (decayed, missing, and filled teeth).

Prior to any invasive intervention during DGA, a detailed screening of the current situation was conducted through examination, photographs, and radiographs. The dental treatment during DGA included conventional restorative procedures, such as composite restoration, fissure sealants, composite strip crowns, pulp therapy, stainless-steel crowns, or extraction if teeth were considered not worth retaining.

### Data collection

To assess the child’s OHRQoL, the validated German version of the ECOHIS was used [11]. The ECOHIS-G questionnaire was self-completed by the participants’ caregiver prior to the DGA (baseline; T0) and 4 weeks later at the post-operative review appointment (T4). The German ECOHIS consists of 13 items and comprises two main subscales, namely, a child impact section and a family impact section. The child impact section is subdivided into four domains: child symptoms (one item), child function (four items), child psychology (two items), and child self-image and social interaction (two items). The family impact section covers two domains: parental distress (two items) and family function (two items) [11]. The questions of the child impact section and family impact section investigate the frequency of an oral health-related problem and are scored on a 5-point Likert scale as follows: never (score 0), hardly ever (score 1), occasionally (score 2), often (score 3), very often (score 4), and don’t know (score 5). The ECOHIS score can be determined by taking the sum of all responses to all 13 items. The child impact score ranges from 0 to 36 and the family impact score from 0 to 16, which results in a total ECOHIS-G score of 0 to 52. The higher the overall sum score, the greater the impact of an oral condition on the child’s OHRQoL.

We also included two additional questions in the questionnaire to evaluate the status of general wellbeing and oral health of the participant. The caregiver was required to answer the questions “How would you rate the general health of your child?” and “How would you rate the oral health of your child?” based on the answer options: excellent (score 1), very good (score 2), good (score 3), fair (score 4), or poor (score 5). For completed questionnaires with up to two missing or “Do not know” responses in the child impact section or one missing item or “Do not know” answer in the family impact section, the score for the missing value was imputed with the average score from the rest of the item section. If more items on a scale were missing or other collected variables were omitted, the data of the participant were excluded.

### Statistical analysis

The data were collected and analysed by SPSS 24.0 (IBM Corp., Chicago, IL). We determined the magnitude of change in OHRQoL after DGA treatment by subtracting the ECOHIS scores at follow-up from those at baseline. The same calculations were made for the child and family sections as well as all the domains of ECOHIS. The Wilcoxon signed-rank test served to compare baseline and follow-up scores regarding statistical significance of potential changes. The effect size was calculated by dividing the mean of change score by the standard deviation of the baseline score [12]. An effect size of < 0.2 indicated a small, but clinically meaningful magnitude of change, 0.2–0.7 a moderate change, and > 0.7 a large change.

## Results

In total, 89 children aged 2 to 5 years and their caregivers were recruited. Nine children were excluded because the pre- or post-operative ECOHIS contained more missing items than allowed. Eighty children were included in our analysis (mean age 3.6 years (± 1.1 years; 42.5% female, 57.5% male), showing a mean dmft of 12.4 (± 6.1) (Table 1).

**Table 1:** General characteristics of participants

Characteristics	Completed questionnaire at follow-up (*N* = 80)	Percentage (%)
Sex (%)		
Male	46	57.5
Female	34	42.5
Mean age (years, SD)	3.6 (1.1)	
Age group (%)		
2 and < 4 years	33	41.3
4 and 5 years	47	58.7
Mean dmft score (SD) Male Female	12.5 (6.1) 13 (6.8) 11.7 (5.2)	

The mean overall ECOHIS scores decreased significantly (*p* < 0.001) after treatment under DGA from 14.60 (± 7.71) to 9.89 (± 6.34), demonstrating a large effect size of 0.8. The scores of the two subscales of the ECOHIS, the child impact section (10.01 [± 6.23] to 6.00 [± 4.15]), and the family impact section (4.56 [± 3.16] to 2.98 [± 2.48]) also decreased significantly (*p* < 0.001) between both time points, demonstrating large (child impact section) and moderate (family impact section) effect sizes, respectively (Table 2).

**Table 2:** Mean ECOHIS scores before treatment at baseline (T0) and at follow-up 4 weeks (T4) after DGA

ECOHIS domain	Pretreatment (T0) Mean (SD)	Posttreatment (T4) Mean (SD)	*p* value	Change score Mean (SD)	Effect size	Description
Total scale	14.60 (7.71)	9.89 (6.34)	< 0.001	5.58 (6.16)	0.8	Large
Child impact section (9) Child symptom Child function Child psychology Child self-image	10.01 (6.23) 2.03 (1.07) 4.61 (1.18) 1.85 (1.12) 1.05 (0.95)	6.00 (4.15) 0.90 (0.79) 3.01 (0.82) 1.30 (0.72) 0.85 (0.60)	< 0.001 < 0.002 < 0.001 < 0.006 < 0.008	4.01 (4.87) 1.12 (1.08) 1.60 (1.13) 0.55 (1.05) 0.20 (0.93)	0.8 1.2 1.6 0.6 0.3	Large Large Large Moderate Moderate
Family impact section (4) Parental distress Family function	4.56 (3.16) 2.51 (1.32) 2.05 (1.07)	2.98 (2.48) 1.51 (0.90) 1.49 (0.83)	< 0.001 < 0.001 < 0.001	1.59 (2.38) 1.00 (1.16) 0.57 (0.99)	0.6 0.9 0.6	Moderate Large Moderate

**Fig. 1:**
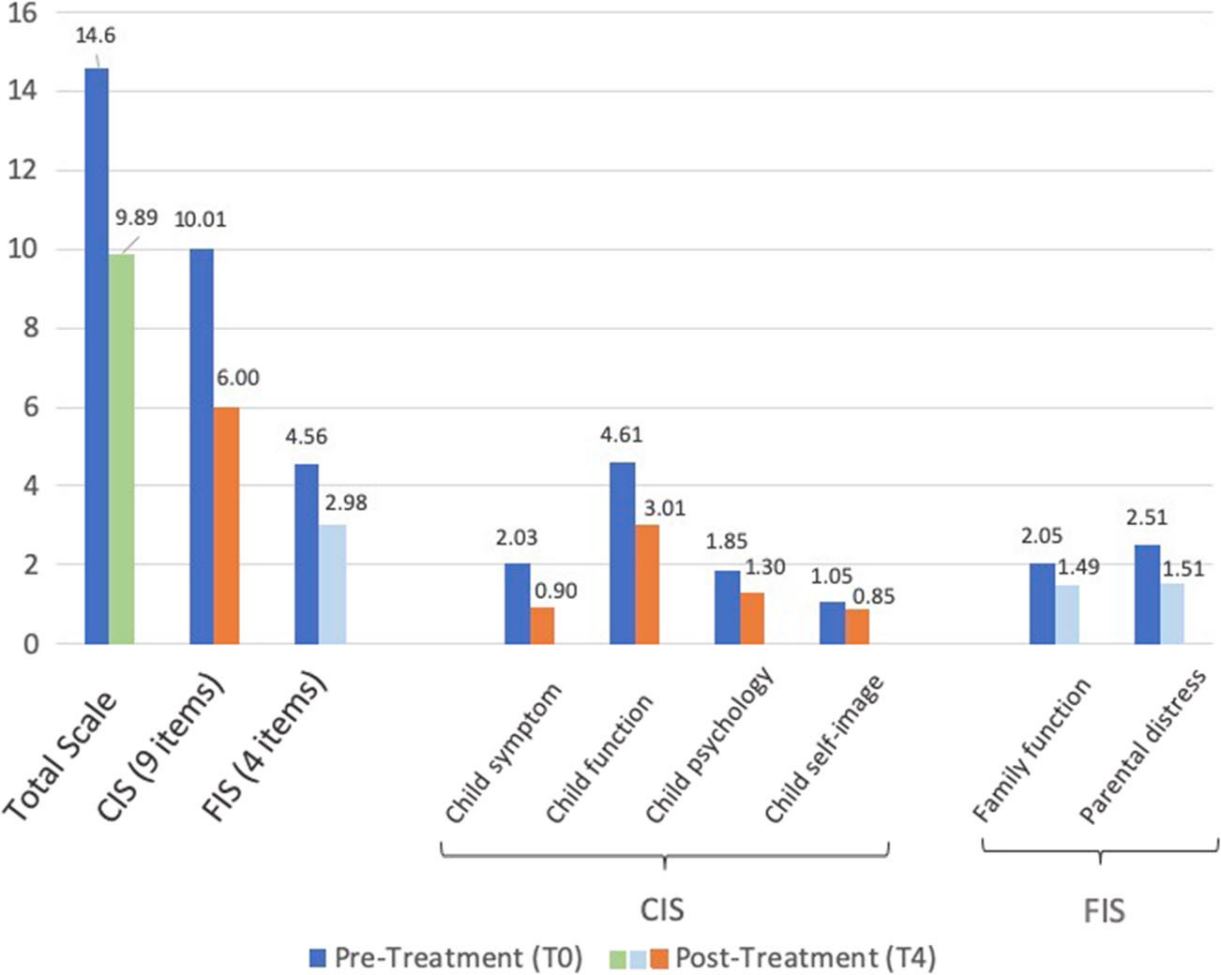
Mean ECOHIS scores before treatment at baseline (T0) and at follow-up 4 weeks (T4) after DGA

In the child impact section, the greatest decrease was found in the domains of child self-image and child psychology (100% and 97.4 %, respectively), whereas the domain of parental distress showed the greatest decrease in the family impact section with 96.2 %. All scores of all items and questions of the child impact section and family impact section showed a significant decrease (*p* < 0.001) and large effect sizes except for the items assessing child self-image (*p* = 0.008) which demonstrates a medium effect size (Table 2). The child symptom and child function domains show a large effect size (1.2 and 1.6, respectively), whereas the child psychology (0.6) and child self-image domains (0.3) only display a moderate effect size (see also Fig. 1 for an overview of the changes of each subscale and domain at baseline (T0) and posttreatment (T4)).

Table 3 presents data on the prevalence of the items on the ECOHIS at baseline and at the 4 weeks follow-up. Before treatment under DGA, “pain in the teeth, mouth, and jaws” and “difficulty eating some foods” were the most frequently reported impacting factors among the children (73.7% and 48.8%, respectively) on the child impact section; on the family impact section, 43.7% and 35.0% of the parents reported “feeling guilty” and “taking time off from work”, respectively. After the treatment, all items improved from before to after the treatment.

**Table 3:** Prevalence of affected ECOHIS items before treatment at baseline (T0) and at follow-up 4 weeks (T4) after DGA

Item	Pretreatment (T0) *N* (%)	Posttreatment (T4) *N* (%)
Child impact section		
Pain in the teeth, mouth, and jaws	59 (73.7)	19 (23.7)
Difficulty drinking hot or cold beverages	28 (35.0)	20 (25.0)
Difficulty eating some foods	39 (48.8)	18 (22.5)
Difficulty pronouncing any words	27 (33.7)	13 (16.2)
Missing preschool, day care, or school	19 (23.7)	11 (13.7)
Trouble sleeping	25 (31.2)	14 (17.5)
Being irritable or frustrated	26 (32.5)	6 (7.5)
Avoided smiling or laughing	9 (11.2)	4 (5.0)
Avoided talking	13 (16.2)	5 (6.2)
Family impact		
Parents being upset	28 (35.0)	10 (12.5)
Parents feeling guilty	35 (43.7)	21 (26.2)
Parents taking time off from work	28 (35.0)	16 (20.0)
Financial impact on the family	19 (23.7)	16 (20.0)

## Discussion

Significant improvements in oral health-related quality of life were observed 4 weeks after DGA in children suffering from ECC. Oral rehabilitation under GA has proven to be an efficient way to treat children with behavioural problems. Instead of delaying treatment due to lack of cooperation, DGA allows dentists to offer significantly improved dental care and immediate pain relief [13]. Our study shows that these procedures lead to positive change in the child’s quality of life, which is evident by the significant decrease in the overall ECOHIS scores and a significant positive impact on the parental emotions and distress after dental treatment under GA. Our study supports the already existing evidence in the literature for a positive impact of GA on the OHRQoL for practitioners who are dealing with little compliant children.

It is well-known that untreated ECC leads to severe chronic pain and overall health restrictions due to undesirable side effects such as oral inflammations [9]. These findings were supported by our study as the majority of children complained about “pain in the teeth, mouth, and jaws” and “difficulty eating some foods”, before dental treatment under GA (73.7% and 48.8%, respectively). Our findings concur with previous studies conducted by Hao-Feng Jiang et al. [14] and Yawary et al. [15], where these domains were also the most frequently reported impacts for children (75.9% and 72.5%).

We observed that the section with the highest improvement was the child psychology section (consists of the subsections “trouble sleeping” and “being irritable and frustrated”, see Table 3), with 63.7% affected children at baseline compared with 25% after DGA. This might be explained by the fact that chronic pain and untreated dental problems can lead to spill-over effects such as sleep deprivation and thus to a more encompassing reduction in a child’s quality of life. The relief that is being accomplished with treatment under GA can, in these cases, go beyond mere pain reduction and have a positive influence on the general psychological state of the child.

An unexpected and in previous studies unobserved result is the moderate effect size of the family impact section. However, a closer look reveals that because there is no significant decrease in the financial impact section (23.7 to 20.0%), this result suggests that the treatment under GA might have been a financial burden on the parents. Given that the costs of GA and certain dental treatment procedures (e.g. SSC, space maintainer) are not covered by health insurance, the resulting tension between the parents’ expectations regarding the financial expenses associated with the treatment and their child’s health might explain this phenomenon. Nevertheless, the overall positive impact shows that the benefits from GA are greater than these possible drawbacks.

Finally, we want to address three possible limitations of this study. The participants for this study were recruited at the Medical University in Vienna and a private practice in Salzburg. It could be argued that local factors might have impacted the baseline scores and the effect size of the OHRQoL being affected by different dmft values in each geographical location. However, the difference in dmft between both locations (12.76 [± 6.5] vs 10.4 [± 2.4]) was not significant. For this reason, we made the simplifying assumption to treat the participants from both locations as a homogenous group.

A part of this study was conducted at the Medical University of Vienna, in the Department of Paediatric Dentistry. Eighty children were successfully followed up through a 4-week post-operative review appointment, which is unlike the more usual 3-month follow-up time in other studies [15–17]. This is due to a structural peculiarity. As the only university clinic throughout Austria, we receive a lot of referred and admitted patients, which return to their dentist after oral rehabilitation under DGA. This makes it difficult to follow up on the patient on a long-term basis and evaluate the long-term effect of oral rehabilitation under DGA. Nevertheless, to prevent a deterioration of the child’s oral health after DGA, we provided information to parents and thorough oral hygiene instruction at every appointment to enable a sufficient improvement in oral health behaviour. Patients that were not referred by their own dentists or those who wanted to continue to be under our care were included in our follow-up program to maintain the oral status after DGA.

As it is with all studies regarding the treatment of ECC in children, a control group to evaluate changes of the ECOHIS scores between children that were treated under DGA and children that were not treated was not included for ethical reasons [18]. Furthermore, investigating how different treatment options under DGA impact the OHRQoL presents an interesting road for further research. Because this requires a different study design, e.g. a randomised controlled trial to assess effects of different interventions in comparison with each other, than the one that was adopted for this study, it was not possible to explore this relationship.

The study closes an important gap in the literature by being the first of its kind in a German-speaking country that assessed the OHRQoL of Austrian preschool children after oral rehabilitation under DGA. This study shows that if ECC is left untreated, children will experience toothache; difficulty in eating, drinking, and sleeping; and thus an overall negatively affected OHRQoL.

In conclusion, the study confirmed that there is a significant improvement in preschool children’s OHRQoL 4 weeks after dental treatment under general anaesthesia. However, there has been little research so far on the impact of different treatment options under GA, so further investigation in this area is needed. Even though these results promote treatment under GA in children, a special focus should be placed on the prevention of dental caries.
